# Dengue Virus Serotype 4, Roraima State, Brazil

**DOI:** 10.3201/eid1710.110776

**Published:** 2011-10

**Authors:** Pablo Oscar Amézaga Acosta, Rodrigo Melo Maito, Fabiana Granja, Joel da Silva Cordeiro, Thalita Siqueira, Mayara Nunes Cardoso, André de Lima Corado, Raphaela Honorato Barletta-Naveca, Felipe Gomes Naveca

**Affiliations:** Author affiliations: Universidade Federal de Roraima, Boa Vista, Brazil (P.A. Amézaga Acosta, F. Granja, J. da Silva Cordeiro, T. Siqueira, M. Nunes Cardoso, A. de Lima Corado);; Laboratório Central de Roraima, Boa Vista (R. Melo Maito);; Instituto Nacional de Pesquisas da Amazônia, Manaus, Brazil (R.H. Barletta-Naveca);; Instituto Leônidas e Maria Deane, Fiocruz, Brazil (F. Gomes Naveca)

**Keywords:** Dengue virus type 4, Brazil, phylogeny, viruses, letter

**To the Editor:** Temporão et al. recently reported the detection and characterization of dengue virus serotype 4 (DENV-4) in Boa Vista, Roraima, Brazil (*1*). To date, 4 subtypes of DENV-4 have been recognized: genotype I, which comprises Asian strains (e.g., Thailand-1978-U18441); genotype II, which has been detected since the early 1980s in the Americas (e.g., Brazil-1982-U18425); genotype III, which comprises recently emerged Thai strains (GenBank accession no. AY618989); and genotype IV, which comprises sylvatic strains (GenBank accession no. EF457906) (*2*).

Temporão et al. conducted phylogenetic analysis of envelope gene sequences and concluded that 3 samples of DENV from Roraima in 2010 were DENV-4, genotype I (*1*). Unfortunately, the authors mistakenly labeled Asian strains (Thailand-1978 and -1985) as genotype II, and American strains (e.g., Brazil-1982) as genotype I. Those DENV-4 strains isolated in Roraima in 2010 in fact belong to genotype II (*2**,**3*). We had previously analyzed 2 samples isolated from Roraima in 2010 by using C/prM nucleotide sequencing and maximum-likelihood phylogenetic reconstruction. Our results, presented at the XXI National Meeting of Brazilian Society for Virology in October 2010, show that both isolates are indeed genotype II (*3*). Nucleotide sequences are available in GenBank under accession nos. HQ822125 and HQ822126.

Temporão et al. also concluded that because only genotype II (reported as genotype I) was identified in their samples, “[it] excludes the possibility that Asian genotypes previously circulated in Brazil.” Beyond its obviously flawed logic, we believe that this statement lacks scientific support; DENV-4 genotype I, closely related to Chinese and Philippine strains, has in fact been shown to occur in the city of Manaus, ≈800 km south of Boa Vista, as reported in 2 recent articles (*4**,**5*). Circulation of DENV-4 genotype I in northern Brazil, probably related to increasingly intense trade with Asian countries, may be sporadic and geographically limited as yet (*5*), but ignoring this evidence can hardly be helpful for dengue surveillance and control.
