# Supplementary material for: Dengue Virus Serotype 4, Roraima State, Brazil
Source: Emerg Infect Dis. 2011 Oct;17(10):1979–81. doi: 10.3201/eid1710.110776 (PMC3310685; doi:10.3201/eid1710.110776)
Supplement: Related texts

# Reply

**Authors:** Pedro F.C. Vasconcelos, Márcio R.T. Nunes

**Affiliations:** Author affiliation: Evandro Chagas Institute, Ananindeua, Brazil

**In Response:** With regard to their comment on our letter, Dengue Virus Serotype 4, Roraima State, Brazil (*1*), Amézaga Acosta et al. correctly refer to an erroneous correlation between genotype I and American strains and between genotype II and Asian strains (*2*). That was a typographic error in the text and figure. Our Figure shows the correct association.

**Figure:** Phylogenetic tree demonstrating the 2 clusters of dengue virus serotype 4 correctly associated with corresponding genotypes: genotype II, American strains, including strains isolated in Roraima in 2010; and genotype I, 2 Asian strains. **Boldface** indicates strains isolated in Roraima. Scale bar indicates 1% nucleotide sequence divergence.

Amézaga Acosta et al. also suggested a scientific inconsistency, that our results exclude circulation of previous Asian strains in Brazil, and argue that Figueiredo et al. (*3*) published a letter on the introduction of DENV-4 in Manaus. However, Amézaga Acosta et al. probably do not know that the article by Figueiredo et al. has been contested by the Brazilian Ministry of Health for failing to demonstrate any irrefutable scientific result, including the virus isolation. Our statement was logically based on strong epidemiologic surveillance, virus isolation, serologic evidence (hemagglutination inhibition assay and immunoglobulin ELISA), and clinical aspects.

Regarding circulation of 2 genotypes in Brazil, when the article was written, epidemiologic and molecular evidence supported the hypothesis of circulation of only the American genotype (II) in northern Brazil and not the Asian (I) and American (II) genotypes at the same time (*1**,**2**,**4*). More specifically to the Manaus finding, no virus was isolated and no strong serologic evidence (in the lack of virus isolation) was provided, and the Ministry of Health considers this article a mistake, probably caused by laboratory contamination with Asian genotype I.

Possible introduction and detection of Asian DENV-4 strains in Brazil should not be ignored because the possibility of multiple introduction events in the country resulting from intense transit of people and commercial activities across Brazil from the Caribbean and Asian regions poses a real risk. However, at this time, only genotype II has been isolated and genetically characterized (*1*). The previously published articles lack strong and reliable scientific evidence.

## References

[1] Temporão JG, Penna GO, Carmo EH, Coelho GE, do Socorro Silva Azevedo R, Nunes MR, Dengue virus serotype 4, Roraima State, Brazil [letter] [PubMed]. Emerg Infect Dis. 2011;17:938–40.2152942110.3201/eid1705.101681PMC3321786

[2] Amézaga Acosta PO, Melo Maito R, Granja F, da Silva Cordeiro J, Siqueira T, Nunes Cardoso M, Dengue virus serotype 4, Roraima State, Brazil [letter]. Emerg Infect Dis. 2011;17:1979–80.2200039610.3201/eid1710.110776PMC3310685

[3] Figueiredo RM, Naveca FG, Bastos MS, Melo MN, Viana SS, Mourão MPG, Dengue virus type 4, Manaus, Brazil. Emerg Infect Dis. 2008;14:667–9. 10.3201/eid1404.07118518394292PMC2570911

[4] Melo FL, Romano CM, Zanotto PM. Introduction of dengue virus 4 (DENV-4) genotype I into Brazil from Asia? PLoS Negl Trop Dis. 2009;3:e390. 10.1371/journal.pntd.000039019399169PMC2669502
